# Traumatic Infection

**Published:** 1896-09

**Authors:** 


**Traumatic Infection.** By CHARLES BARRETT LOCKWOOD. Pp. 138. Edinburgh: Young J. Pentland. 1896.

The book is a reprint of the Hunterian lectures delivered at the Royal College of Surgeons last year. It deals exhaustively with the various forms of micro-organisms, which produce such diseases as septicæmia and septic peritonitis, their modes of infection, and the result of their growth in the tissues. A case of angina Ludovici, in which a mixed infection of a very complicated kind was found is recorded, and the very interesting question of the relation of hectic fever to septicæmia is discussed. The work is well illustrated with pictures of micro-organisms in the tissues. As a record of much laborious original investigation, and as a reference to the work of others in the same department of study, the book is of great value.

